# Abstract of the Proceedings of the Buffalo Medical Association

**Published:** 1856-01

**Authors:** James M. Newman


### ART. IV.—*Abstract of the Proceedings of the Buffalo Medical Association.*

TUESDAY EVENING, Dec. 4, 1855.

Association met, Tuesday evening, Dec. 4th, at their room South Division street.

Present—Drs. Wilcox, Gould, Root, Nelson, Hutchins, Samo, Miner, Newman, Hunt and White.

The President being absent, Dr. Nelson was chosen to preside.

The Secretary being also absent at the opening of the meeting, Dr. Newman was elected secretary *pro tem.*

The minutes of the previous meeting were read and approved.

*Dr. Nelson* then related the following case:

CASE. On Saturday, Dec. 1, 1855, I was requested to see a lady who it was supposed had a needle imbedded in the left thigh. On examination I found a foreign substance with a sharp point, like the broken eye of a needle, presenting in the superior portion of Scarpa's space; the lady could not recollect ever having been injured by a needle. I accordingly cut down, and after a little difficulty, succeeded in removing, not a needle, but a small piece of bone of about the shape and size of a grain of wheat. The lady then gave me the following statement of facts: Twenty-two years ago, when she was a child of six years of age, she was riding home from her father's orchard sitting on a basket of fruit in the wagon, when suddenly both she and basket fell off. It was not supposed, at that time, she was hurt, though she complained slightly of the left knee; some few days afterward in going into the store-room, she again struck the same knee against a box, which gave her very much pain, so much so that it was necessary to send for a physician, who, on examining it, declared there was nothing the matter with it. From that time till she was 15 years of age, she would occasionally experience severe pain in that knee, extending both up and down the limb, which caused her friends much anxiety, and subjected her frequently to a medical examination, without any satisfactory result. Two years ago a foreign substance was felt deep in the middle and inside of the thigh, which up to the time of operating, had ascended a space of two inches, and latterly had interfered with her walking, which was the reason of having it removed. I have since examined it, with the assistance of Doctors Rochester and Boardman, under the microscope, and these gentlemen coincide with me that it is bone.

*Remarks.* We know whither it went, but "whence came it?" is the question; could it have been a small portion of the inner part of the patella? if so, does it not elucidate a most extraordinary fact how foreign bodies will sometimes travel a very great distance to gain the surface, instead of the usual routine of inflammation and suppuration, for had the usual symptoms of such an accident, inflammation, suppuration and exit of the broken piece of bone from the abscess occurred, the most likely result would have been disease of the knee-joint or anchylosis.

*Dr. Hutchins* reported the following case of morbid developments, in the person of a patient who first came under his notice a week since. The case consists in the successive development of tumors in different parts of the body, which have already reached a large size, and still manifest a disposition to increase.

The first tumor made its appearance some few months since in the left groin. It now extends from on a line with the anterior superior spinous process, down the groin into the scrotum of the left side. The leg is enormously swollen, apparently from obstruction to the circulation. He has a tumor, also, in the hollow of the same foot, and one behind the left ear. These tumors are insensible to the touch, and he has never had pain in them, or at least up to to-day, when for the first time they have exhibited some tenderness. The appetite is good and the patient feels well. He had already before coming under the notice of Dr. H., been under the care of a physician who had apparently exhausted every remedy supposed to exert any influence upon the glandular system; but they have nevertheless continued constantly to increase.

*Dr. Root* stated that he now had a case of small-pox in the hospital, being the first case there had been in the house since July last. The patient is a boatman upon the lakes. The long entire exemption of our city from the presence of variolous disease, was worthy of remark.

*Dr. Root* inquired as to the constancy of bloody expectoration in pneumonia. Whether bloody sputa was not considered pathognomic, and as being invariably present in the disease in adults? He was called on Saturday morning last to a patient taken sick the previous day with pneumonia. There is solidification of the lower lobe of the right lung; there is and has been no bloody expectoration. The patient is a female, aged twenty-two or twenty-three years.

The experience of the association was the uniform appearance of the characteristic expectoration, especially in adults.

*Dr. White* wished to remark upon what appeared to him the unusual frequency of carbuncle. He has seen six or eight cases within the last fortnight: has seen two to-day. He has seen more cases within the last twelve months, than during the whole of the last twenty-three years; and what was, perhaps, worthy of remark, the frequency of those cases in young persons, and otherwise in good health.

Most of the members present bore testimony to their frequency in their practice, and Dr. Hutchins related a case where carbuncle followed the application of cups used for the treatment of lumbago, and occupying the exact spot covered by the cups.

*Dr. Hunt* gave a brief report of two cases of pneumonia. One of these presented no especial point of interest, except that it was a sthenic pneumonia, in a robust and healthy laborer, and occupied the lower lobe of the right lung; terminating by resolution under the treatment by diaphoretics and blistering, only. The period of attendance was five days. The whole duration of disease up to time of discharge, about ten days.

In the second case, by a curious chain of accidents, several facts were developed as to the tolerance of tonics in acute sthenic pneumonia; and, finally, an opportunity afforded to witness the post-mortem appearances of a lung yet in the first stage of pneumonia.

The patient, a young French girl of full figure, who had a few weeks previously had intermittent fever, and, subsequently to that, a laryngitis of mild type, but who was then in pretty good health, had a severe chill while in church. The young gentleman under whose immediate charge she fell, seeing her in the chill, very naturally considered it a recurrence of the intermittent, and prescribed quinine in five grain doses, to be taken every four hours during the succeeding day (Monday.) She was not confined to the bed on Tuesday, but on Wednesday I saw her, and detected a pneumonia of the lower lobe of the right lung, the symptoms being severe cough with viscid expectoration, and slight dullness with very distinct crepitant râle over the whole affected region. She took liquor ammon. acet. every hour during the day, and a ten grain Dover's powder at night. The next day the pulse was about 90, and soft; skin moist; cough less severe; sounds of lung the same. Treatment was continued.

On Friday the sounds of the lung indicated (together with the general symptoms) a termination by resolution.

The treatment, viz., liquor ammon. acet. every two hours, and a ten grain Dover's powder at night, was continued. The febrile symptoms being abated, a blister was placed over the lower right lobe, posteriorly.

Saturday, 11, A. M. Found my patient moribund. The skin was cool, clammy, and deeply cyanosed from imperfect æration; the extremities cold; the pulse slow, laboring, but not full; the respiration occurring only at long intervals, and then rattling and gasping; and sounds of lung not distinguishable. Profound coma closes the catalogue of symptoms.

I learned that during the night she had been delirious, growing gradually more frantic until it had been difficult to control her, that on the night before that some delirium had followed the Dover's powder, and that her present condition had come on rapidly. She lived a few hours longer, her system not responding to remedies.

So unexpected a result made a post-mortem examination desirable, which was had the next day, twenty hours after death. Present, Drs. Rochester, Lemon, and myself. The following results were obtained:

*Abdomen*—healthy in all its viscera.

Larynx—entirely healthy; left lung healthy throughout without even hypostatic congestion; while the two upper lobes of the right luug were equally clear from disease.

The lower lobe of the right lung was not solidified, but was nevertheless heavier than natural, presenting a bright red uniform color, due to congestive action. The substance of the lung cut smooth and waxy, but was, nevertheless, to a degree crepitant throughout.

It was then evident that she had simple pneumonia of the lower lobe, of a mild type, which at the moment of death was probably progressing toward a cure. No cause for death was found in the examination.

Various circumstances, which it would, perhaps, be improper to repeat here, led to the conviction in the minds of all interested, that she died from overdoses of some form of opiate, administered either by mistake, or in an officious and unauthorized attempt to quiet her delirium.

Recurring to the beginning of the disease, the neutral, or at any rate not injurious, effect of the quinia upon an incipient pneumonia, is especially worthy of notice.

Adjourned. JAMES M. NEWMAN, Sec. *pro. tem.*

